# Disparities in Diabetes Deaths Among Children and Adolescents — United States, 2000–2014

**DOI:** 10.15585/mmwr.mm6619a4

**Published:** 2017-05-19

**Authors:** Sharon Saydah, Giuseppina Imperatore, Yiling Cheng, Linda S. Geiss, Ann Albright

**Affiliations:** 1 National Center for Chronic Disease Prevention and Health Promotion, Division of Diabetes Translation, CDC.

Diabetes is a common chronic disease of childhood affecting approximately 200,000 children and adolescents in the United States (*1*). Children and adolescents with diabetes are at increased risk for death from acute complications of diabetes, including hypoglycemia and diabetic ketoacidosis (*2*,*3*); in 2012, CDC reported that during 1968–2009, diabetes mortality among U.S. persons aged ≤19 years declined by 61% (*4*). CDC observed disparities by race during 1979–2004, with black children and adolescents dying from diabetes at twice the rate of white children and adolescents (*5*). However, no previous study has examined Hispanic ethnicity. CDC analyzed data from the National Vital Statistics System for deaths among persons aged 1–19 years in the United States during 2000–2014, with diabetes listed as the underlying cause of death overall, and for Hispanic, non-Hispanic white (white), and non-Hispanic black (black) children and adolescents. During 2012–2014, black children and adolescents had the highest diabetes death rate (2.04 per 1 million population), followed by whites (0.92) and Hispanics (0.61). There were no statistically significant changes in diabetes death rates over the study period, but disparities persisted among racial/ethnic groups. Death from diabetes in children and adolescents is potentially preventable through increased awareness of diabetes symptoms (including symptoms of low blood sugar), earlier treatment and education related to diabetes, and management of diabetes ketoacidosis. Continued measures are needed to reduce diabetes mortality in children and understand the cause of racial and ethnic disparities.

Diabetes mortality among persons aged 1–19 years during 2000–2014 was examined using information from death certificates filed in all 50 states and the District of Columbia (DC) and collected by CDC’s National Center for Health Statistics. Hispanic ethnicity was collected on death certificates for all 50 states and DC starting in 1997. A diabetes death was defined as one with an *International Classification of Diseases, Tenth Revision* underlying cause of death code of E10–E14. Annual U.S. Census estimates for persons aged 1–19 years (https://wonder.cdc.gov/wonder/help/cmf.html#Population) were used as the denominators. Mortality estimates were obtained from the CDC Wonder online database (https://wonder.cdc.gov/mortSQL.html). To produce stable mortality estimates, diabetes death rates were analyzed in 3-year intervals. Infants (children aged <1 year) were excluded because methods for calculating neonatal and postnatal mortality rates differ from those for children aged ≥1 year. Race/ethnicity was categorized into the following groups: non-Hispanic black (black), non-Hispanic white (white), Hispanic, and all races/ethnicities (all children and adolescents). Hispanic includes all Hispanic origins, and persons who are Hispanic can be of any race. There were too few deaths among the other race/ethnicity groups to produce reliable estimates for those groups.

Joinpoint regression was used based on 3-year intervals to analyze trends using Hudson’s algorithm, which includes time as a continuous variable (*6*). Joinpoint regression uses permutation tests to identify points where linear trends change significantly in direction or magnitude (i.e., joinpoints). The rate of change was tested for each trend to determine whether it was significantly different from zero, and each trend was described in the final model by an annual percentage change with a 95% confidence interval (CI). The National Cancer Institute’s Joinpoint software was used (https://surveillance.cancer.gov/joinpoint/). Rate ratios and 95% CIs were calculated to compare racial/ethnic groups in each 3-year interval.

The total number of deaths from diabetes among all U.S. persons aged 1–19 years decreased from 265 (1.15 per 1 million) during 2000–2002 to 228 (0.97 per 1 million) during 2012–2014 (Table) (Figure). During 2012–2014, the highest diabetes death rates in this age group (2.04 per 1 million population) was among blacks, and the lowest was among Hispanics (0.61); death rates among whites were intermediate between blacks and Hispanics (0.92). From 2000–2002 to 2012–2014, the annual percentage change in diabetes death rate among all children and adolescents was -1.7%. From 2000–2002 to 2012–2014, the annual percentage change was 0.6% among Hispanics, -2.9% among blacks, and -0.92% among whites. None of these changes was significantly different from zero. There were no significant joinpoints, consistent with a straight line.

**TABLE:** Deaths from diabetes per 1 million persons aged 1–19 years, by race and ethnicity and race/ethnicity rate ratios — United States, 2000–2014

Characteristic	No. (95% CI)					Absolute change (95% CI)*	Annual percentage change (95% CI)
	2000–2002	2003–2005	2006–2008	2009–2011	2012–2014		
**Total no. deaths**	**265**	**285**	**251**	**231**	**228**	-**37**	**—**
Deaths and rates of death							
All racial/ethnic groups^†^	1.15 (1.01 to 1.29)	1.22 (1.08 to 1.36)	1.06 (0.93 to 1.19)	0.97 (0.85 to 1.1)	0.97 (0.84 to 1.1)	0.18 (-0.38 to 0.02)	1.67 (-3.39 to 0.09)
Hispanic	0.65 (0.42 to 0.95)	0.52 (0.33 to 0.78)	0.69 (0.48 to 0.96)	0.73 (0.52 to 0.99)	0.61 (0.42 to 0.85)	-0.04 (-0.04 to 0.28)	0.63 (-4.08 to 5.57)
Black	2.39 (1.91 to 2.95)	2.72 (2.21 to 3.32)	2.26 (1.80 to 2.80)	1.43 (1.07 to 1.88)	2.04 (1.60 to 2.57)	-0.35 (-1.04 to 0.35)	-2.89 (-9.17 to 3.83)
White	1.01 (0.85 to 1.18)	1.10 (0.93 to 1.27)	0.97 (0.81 to 1.14)	1.01 (0.84 to 1.18)	0.92 (0.75 to 1.08)	-0.10 (-0.33 to 0.14)	-0.92 (-2.82 to 1.02)
Rate ratios (95% CI)^§^							
Black to white	2.36 (1.80 to 3.08)^§^	2.47 (1.92 to 3.19)^§^	2.32 (1.76 to 3.05)^§^	1.42 (1.03 to 1.95)^§^	2.22 (1.66 to 2.98)^§^	—	—
Black to Hispanic	3.69 (2.38 to 5.73)^§^	5.26 (3.41 to 8.30)^§^	3.28 (2.20 to 4.89)^§^	1.97 (1.30 to 2.98)^§^	3.36 (2.24 to 5.04)^§^	—	—
White to Hispanic	1.57 (1.03 to 2.38)^§^	2.13 (1.37 to 3.30)^§^	1.41 (0.97 to 2.06)	1.39 (0.98 to 1.99)	1.51 (1.03 to 2.21)^§^	—	—

**Abbreviation:** CI = confidence interval.

* From 2000–2002 to 2012–2014.

^†^ Hispanic persons can be of any race; black and white refer to non-Hispanic persons.

^§^ Rate ratio is statistically significantly different from 1.0.

**FIGURE:**
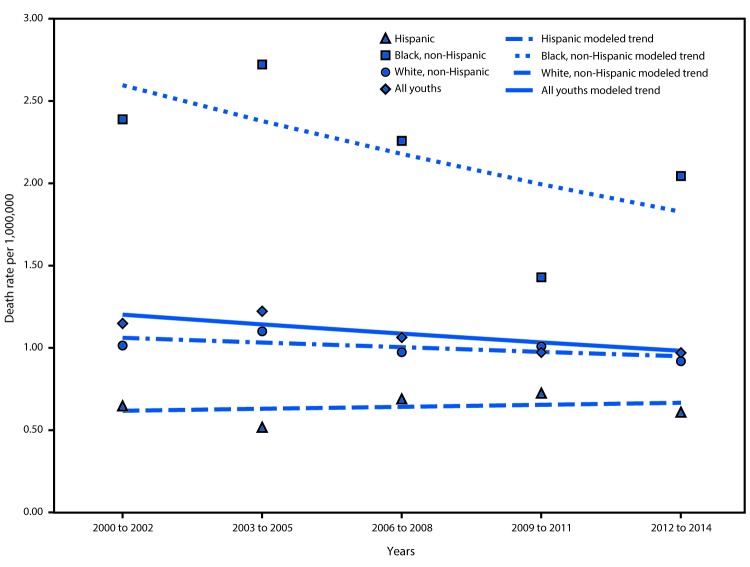
Three-year annual average diabetes death rates* per 1 million among persons aged 1–19 years, by race/ethnicity — United States, 2000–2014 * Symbols indicate observed points; lines indicate modeled trends. There were no significant modeled joinpoints, which is consistent with a straight line

Although there were no statistically significant changes in diabetes death rates from 2000–2002 to 2012–2014, disparities persisted among racial/ethnic groups. During 2000–2002, the diabetes death rate ratio for blacks compared with whites was 2.36 and for blacks compared with Hispanics was 3.69 (Table). This disparity was still present during 2012–2014, when the diabetes death rate for blacks was 2.22 times that of whites and 3.36 times that of Hispanics. Hispanics had the lowest diabetes death rates during all periods. Diabetes death rate for whites was 1.57 (95% CI = 1.03, 2.38) and 1.51 (95% CI = 1.03, 2.20) times that of Hispanics during 2000–2002 and 2012–2014, respectively.

## Discussion

During 2012–2014, among U.S. persons aged 1–19 years, 228 diabetes-related deaths (approximately one per 1 million population) occurred. It is encouraging that, despite increases in diabetes prevalence and incidence among children and adolescents during the 14 years from 2000 to 2014, there was no significant increase in diabetes mortality. However, significant racial/ethnic disparities in diabetes deaths among persons aged 1–19 years persisted. In particular, the death rates among blacks remained approximately twice as high as those of whites and Hispanics, whereas Hispanics had the lowest rates of diabetes mortality during all periods. Among children and adolescents, diabetes deaths are likely caused by acute complications of diabetes (*3*). Therefore, it would be expected that the highest diabetes-associated mortality would occur among racial/ethnic groups with the highest diabetes incidence and prevalence. The incidence of type 1 diabetes for children and adolescents was higher among whites than among blacks in 2011 (*7*), and the prevalence of childhood and adolescent diabetes among whites was higher than among blacks during this same period (*8*). In contrast, this analysis found that diabetes mortality was higher among black children and adolescents than among whites. Reasons for these disparities in deaths from diabetes are likely complex. Possible explanations could include differences in access to health care, health services, diabetes self- and parent-management education, and diabetes care (*2*,*9*,*10*).

The findings in this report are subject to at least three limitations. First, because the small number of diabetes deaths precluded more detailed analysis, multiple years of deaths were combined for reliable estimates, which made it difficult to discern subtle changes in trends. Whereas a previous report observed an increase in diabetes mortality among persons aged 10–19 years (*4*), the small number of deaths in some racial/ethnic groups prevented stratification of the results by age. However, over the 2000–2014 period, only 14% of the deaths occurred among children aged 1–9 years (data not shown). The small number of deaths also precluded analysis by Hispanic subgroups. Second, it is also not known whether diabetes death rates differed by diabetes type. Type 1 diabetes is the most common diabetes type among children and adolescents, and its prevalence varies by race/ethnicity: among persons aged 10–19 years with diabetes, 94.5% of whites, 62.4% of blacks, and 64.8% of Hispanics have type 1 diabetes (*8*). However, in this study, information on the death certificate indicating diabetes type was only available for 24% of all diabetes deaths among persons aged 1–19 years from 2000 to 2014, precluding analysis by diabetes type. Finally, there is a potential for misclassification of race/ethnicity on the death certificate.

This is the first time diabetes mortality among Hispanic children and adolescents has been reported and compared with mortality among whites and blacks. The findings indicate that although the diabetes mortality among children and adolescents has not changed significantly in the United States, disparities by race/ethnicity persist and warrant further research and investigation so that targeted interventions for prevention of diabetes deaths among children and adolescents can be developed and implemented.

**Summary**

**What is already known about this topic?**

Diabetes in children and adolescents is a serious chronic disease. Young persons with diabetes are at risk for death from acute complications of the disease.

**What is added by this report?**

In this first report of diabetes mortality among Hispanic persons aged 1–19 years and comparison with mortality among white and black children and adolescents, there were no statistically significant changes in diabetes death rates from 2000–2002 to 2012–2014. Despite the higher prevalence and incidence of reported diabetes among whites than among blacks, blacks had approximately a twofold increased risk for diabetes death compared with whites and over a threefold increased risk compared with Hispanics.

**What are the implications for public health practice?**

Deaths from diabetes in young persons are potentially preventable. The continued existence of racial/ethnic disparities in diabetes mortality in this age group adds information about Hispanics. Further research to identify health care factors and behaviors that contribute to diabetes mortality in children and adolescents might be helpful in understanding the reasons for disparities by race/ethnicity and focusing future prevention efforts.
